# Supplementary material for: Empirical methods for controlling false positives and estimating confidence in ChIP-Seq peaks
Source: BMC Bioinformatics. 2008 Dec 5;9:523. doi: 10.1186/1471-2105-9-523 (PMC2628906; doi:10.1186/1471-2105-9-523)
Supplement: Additional File 1 — A variety of html documents from the USeq web site detailing the available applications, their best usage, output file type descriptions, command line menus, etc. [file 1471-2105-9-523-S1.zip › USeqUserGuides/JavaDoc.zip!/JavaDoc/trans/bkgrnd/package-summary.html]

trans.bkgrnd


|  |  |  |
| --- | --- | --- |
| **Overview** \| **Package** \| Class \| **Use** \| **Tree** \| **Deprecated** \| **Index** \| **Help** | |  |
| **PREV PACKAGE**   **NEXT PACKAGE** | **FRAMES**    **NO FRAMES**     **All Classes** |  |


---

## Package trans.bkgrnd

For examining background GC content and ratio bias.

**See:**
  
          **Description**

| **Class Summary** | |
| --- | --- |
| **BackGroundOligo** | A control oligo spotted multiple times on the array. |
| **CalcBkgrndRatios** | Calculates a correlation coefficient between ratio scores and tm or gc for a set of CelMapper converted cel/bpmap files. |
| **CalculateBackgroundStats** | For each CelMapper converted cel/bpmap files, this calculates a correlation coeff between pm:mm, pm:tm, pm:gc. |
| **LoadedOligo** | BPMap line contain gc and tm calculations. |
| **MapControlIntensities** | Loads a bpmap file with oligo intensity measurements from multiple '.cel' files after median scaling to 50. |

## Package trans.bkgrnd Description

For examining background GC content and ratio bias.

---


|  |  |  |
| --- | --- | --- |
| **Overview** \| **Package** \| Class \| **Use** \| **Tree** \| **Deprecated** \| **Index** \| **Help** | |  |
| **PREV PACKAGE**   **NEXT PACKAGE** | **FRAMES**    **NO FRAMES**     **All Classes** |  |


---
